# Supplementary material for: Causal Relationship between Aging and Anorexia Nervosa: A White-Matter-Microstructure-Mediated Mendelian Randomization Analysis
Source: Biomedicines. 2024 Aug 16;12(8):1874. doi: 10.3390/biomedicines12081874 (PMC11351342; doi:10.3390/biomedicines12081874)
Supplement: Supplementary file 1 [file biomedicines-12-01874-s001.zip › Supplementary Figure/Figure S4/Singe SNP plot of MR analysis for TL to ubm-b-1885.pdf]

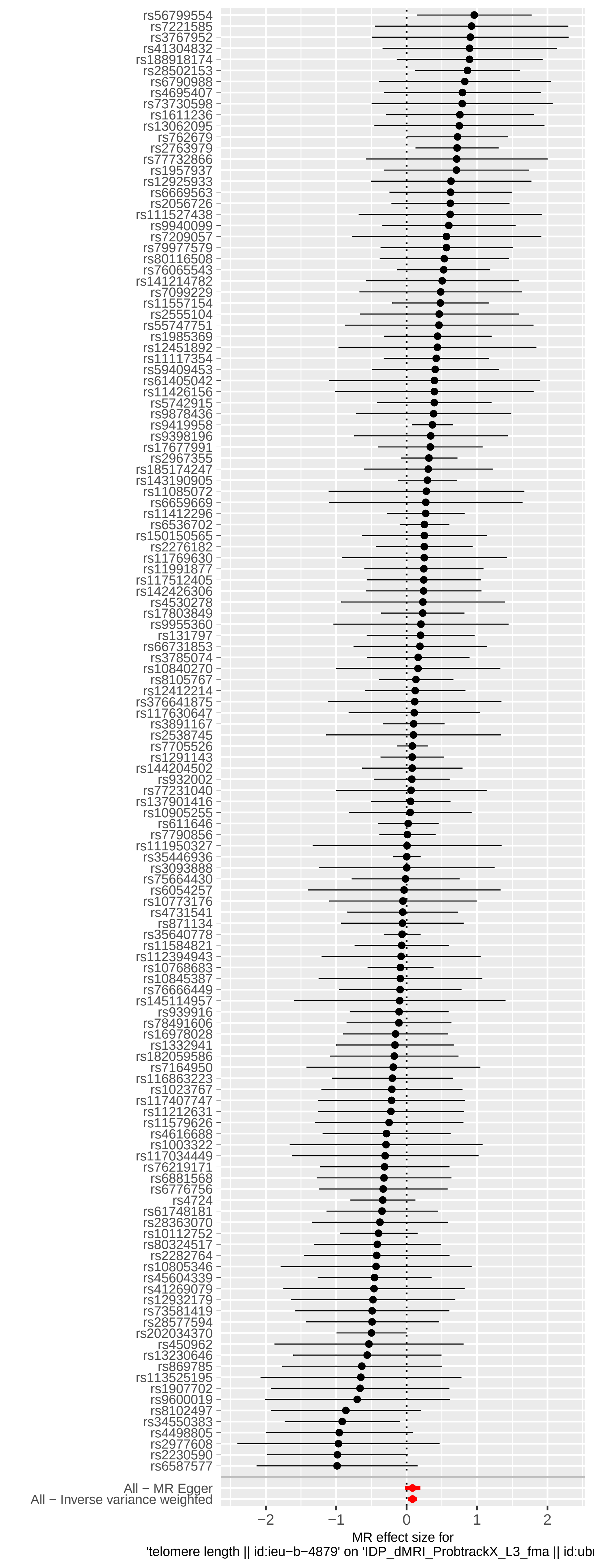
rs56799554
rs7221585
rs3767952
rs41304832
rs188918174
rs28502153
rs6790988
rs4695407
rs73730598
rs1611236
rs13062095
rs762679
rs2763979
rs77732866
rs1957937
rs12925933
rs6669563
rs2056726
rs111527438
rs9940099
rs7209057
rs79977579
rs80116508
rs76065543
rs141214782
rs7099229
rs11557154
rs2555104
rs55747751
rs1985369
rs12451892
rs11117354
rs59409453
rs61405042
rs11426156
rs5742915
rs9878436
rs9419958
rs9398196
rs17677991
rs2967355
rs185174247
rs143190905
rs11085072
rs6659669
rs11412296
rs6536702
rs150150565
rs2276182
rs11769630
rs11991877
rs117512405
rs142426306
rs4530278
rs17803849
rs9955360
rs131797
rs66731853
rs3785074
rs10840270
rs8105767
rs12412214
rs376641875
rs117630647
rs3891167
rs2538745
rs7705526
rs1291143
rs144204502
rs932002
rs77231040
rs137901416
rs10905255
rs611646
rs7790856
rs111950327
rs35446936
rs3093888
rs75664430
rs6054257
rs10773176
rs4731541
rs871134
rs35640778
rs11584821
rs112394943
rs10768683
rs10845387
rs76666449
rs145114957
rs939916
rs78491606
rs16978028
rs1332941
rs182059586
rs7164950
rs116863223
rs1023767
rs117407747
rs11212631
rs11579626
rs4616688
rs1003322
rs117034449
rs76219171
rs6881568
rs6776756
rs4724
rs61748181
rs28363070
rs10112752
rs80324517
rs2282764
rs10805346
rs45604339
rs41269079
rs12932179
rs73581419
rs28577594
rs202034370
rs450962
rs13230646
rs869785
rs113525195
rs1907702
rs9600019
rs8102497
rs34550383
rs4498805
rs2977608
rs2230590
rs6587577
All − MR Egger
All − Inverse variance weighted
−2
−1
0
1
2
MR effect size for
'telomere length || id:ieu−b−4879' on 'IDP_dMRI_ProbtrackX_L3_fma || id:ub
